# Using a Novel Whole Slide Imaging Software Platform for an International Multicenter Validation Study to Assess the Histological Growth Pattern of Liver Metastases

**DOI:** 10.1155/2014/812391

**Published:** 2014-12-30

**Authors:** Yves Sucaet, Wim Waelput, Peter Vermeulen, Gert Van den Eynden

**Affiliations:** ^1^Pathomation BVBA, Prince J. Charlottelaan 10, 2600 Berchem, Belgium; ^2^Department of Pathology, GZA Hospitals, Oosterveldlaan 24, 2610 Wilrijk, Belgium

## Background

The histological growth pattern (HGP) of solid tumor metastases to liver is an easy-to-assess and integrative histopathological parameter of tumor-stromal interactions [1]. With preliminary data suggesting that the HGP of colorectal cancer liver metastases has prognostic value [2], we hypothesize that it also might predict response to therapy [1, 3]. To enhance pathological assessments, we organized an international multicenter validation study within the Liver Metastasis Research Consortium.

## Methods

**Figure 1:**
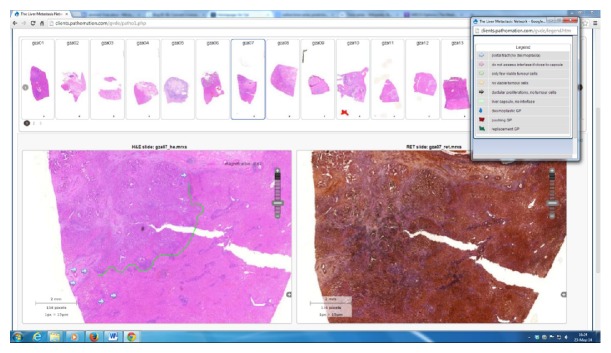


The study design had to offer each participant the opportunity to study a set of reference slides, as well as to score two validation sets. Due to geographic and timing challenges, the study was organized using a web-based digital pathology platform. It consisted of three phases. In Phase 1, the training phase (see Figure 1), participants were instructed in the criteria, guidelines, and pitfalls of the HGP assessment, using hematoxylin-eosin and Gordon Sweet's Silver stained slide of a set of cases selected by two expert pathologists (G. V. den Eynden and P. Vermeulen). In Phase 2, the validation phase, interobserver variability and reproducibility were assessed using a hematoxylin-eosin stained slide of another set of cases. Finally, in Phase 3, the role of Gordon Sweet's Silver staining was determined for the assessment of a third set of cases, to see if this laborious and difficult staining procedure is even necessary.

## Results

25 researchers (pathologists, clinicians, and scientists) at 9XX centers in 8 countries were involved. Case slides were scanned on a variety of scanner platforms and in different locales, so a format-agnostic and vendor/hardware-independent solution was sought. Pathomation (Berchem, Belgium) was chosen as the ideal environment, being able to read all file formats used in the study (NDPI, VSI, SVS, and MRXS). This also permitted us to place custom annotations at the top of each slide, with access rights allowing who could and could not make modifications. The PathoCore product was used to host slides, manage users, and access rights, as well as monitoring. This server software also allowed us to design particular questionnaires and capture responses. The SymPatho viewer was used for the study organizers to provide on-slide digital annotations pertaining to the study slides. Finally, the EmPatho user control was embedded into the LMRN website to display slides and provide a seamless user experience (very important for a person who is not familiar with “digital” pathology). The consortium's website is at http://www.lmrn.org/, and the validation study is at http://clients.pathomation.com/lmrn/.

## Conclusions

Using whole slide imaging and web-based digital pathology facilitates the organization of international multicenter studies involving histological tissue slide assessments. A new user-friendly, format-agnostic, and vendor/hardware-independent platform developed by Pathomation seems very well suited for this.

## References

[1] Van Den Eynden G. G., Majeed A. W., Illemann M. (2013). The multifaceted role of the microenvironment in liver metastasis: biology and clinical implications. *Cancer Research*.

[2] van den Eynden G. G., Bird N. C., Majeed A. W., van Laere S., Dirix L. Y., Vermeulen P. B. (2012). The histological growth pattern of colorectal cancer liver metastases has prognostic value. *Clinical & Experimental Metastasis*.

[3] Vermeulen P. B., Colpaert C., Salgado R. (2001). Liver metastases from colorectal adenocarcinomas grow in three patterns with different angiogenesis and desmoplasia. *Journal of Pathology*.

